# Unmet needs, quality of life and support networks of people with dementia living at home

**DOI:** 10.1186/1477-7525-8-132

**Published:** 2010-11-12

**Authors:** Claudia Miranda-Castillo, Bob Woods, Kumari Galboda, Sabu Oomman, Charles Olojugba, Martin Orrell

**Affiliations:** 1 School of Psychology, Universidad de Valparaíso, Av. Brasil 2140, Valparaíso, Chile; 2 Department of Mental Health Sciences, University College London, 67-73 Riding House Street, London, W1W 7EJ, UK; 3 Dementia Services Development Centre, Bangor University, Neuadd Ardudwy, Holyhead Road, Bangor, LL57 2PX, UK; 4 North East London NHS Foundation Trust, Mascalls Park, Mascalls Lane, Brentwood, Essex, CM14 5HQ, UK; 5 5 Boroughs Partnership NHS Trust, Station Road, Huyton, Liverpool, Merseyside, L36 4HU, UK

## Abstract

**Background:**

There is lack of evidence about the unmet needs of people with dementia (PWD) living at home and the predictors of high levels of unmet needs. The main aim of this study was to identify the relationship between unmet needs, social networks and quality of life of PWD living at home.

**Methods:**

One hundred and fifty two community dwelling PWD and 128 carers were interviewed about PWD's needs, social networks, quality of life and other functional and psychological factors. All the interviews with PWD were carried out at their homes. Interviews with carers were undertaken either at PWD's home, their own home or at the health centre. Whenever possible, PWD and carers were interviewed separately. The data collection took place between November 2005 and July 2007. The majority of participants (129, 84.9%) were recruited from National Health Services (NHS) and the rest (23, 15.1%) were recruited from other organisations such as social services and voluntary organizations in the UK.

**Results:**

The most frequent unmet needs for PWD were daytime activities (77, 50.7%), company (60, 39.5%), and help with psychological distress (47, 30.9%). Higher number of behavioural and psychological symptoms, low-community involvement social networks, having a younger carer and higher carer's anxiety were found to be predictors of higher unmet needs in PWD. Social networks and behavioural and psychological symptoms had an indirect effect on PWD's self-rated quality of life through unmet needs.

**Conclusions:**

Interventions aiming to reduce unmet needs, through the treatment of behavioural and psychological symptoms and the involvement of PWD in the community, would potentially improve PWD's quality of life.

## Background

Unmet needs in PWD have been found to be associated with higher anxiety, depression, and challenging behaviours in care homes [1] as associated with being older, cognitive impairment and living alone in the community [2]. Hoe et al. [3] found that, in care homes, higher quality of life rated separately by residents and staff was associated with fewer unmet needs in residents. However, this relationship has not been studied in a community sample. Evidence shows that PWD with limited social networks are more vulnerable and at risk. Wenger [4] found that the most common social network types in PWD were family-dependent (30%), which is reliant upon a few family members with few friends or other community contacts; and private-restricted (26%), which had small social networks, with very few local kin, friends or other community contacts. Wilcox et al. [5] also found that low level of social support was associated with high score in overall need. It has also been found that PWD with higher unmet needs reported by their carers are more likely to either be placed in a care home or die [6]. This is the first published study to investigate the relationship between unmet needs, social networks and quality of life in PWD living at home aiming to identify factors that are associated with and/or predict unmet needs which could allow, ultimately, the implementation of interventions aimed to reduce unmet needs of PWD. Unmet needs may be associated with lower quality of life [3,7] and smaller social networks [5,8] but in dementia the relationship between social networks and quality of life has not been studied previously. We hypothesize that larger social networks will be associated with better quality of life and that the relationship between social networks and quality of life will be mediated by unmet needs.

## Methods

### Sample

The study design was a cross sectional survey. A total of 152 PWD living at home and their 128 informal carers were recruited from health and social services, and voluntary organisations in North East London (65 PWD and 54 carers), Cambridgeshire (81 PWD and 68 carers) and Liverpool (6 PWD and 6 carers). Inclusion criteria for PWD were: being aged 60 or over, having a diagnosis of dementia according to DSM-IV and living at home (not in institutions). A person was considered an informal carer (spouse, relative, friend or neighbour) when he/she was knowledgeable about the person with dementia and spent a minimum of 4 hours a week in direct contact (face to face) with them. Only one person was considered the nominated carer. A sample, similar to those obtained from previous community studies of dementia in terms of living situation, was recruited: 65% of participants living with another person and 35% living alone [9,10].

At each centre, the manager or appropriate member of staff was requested to make a first approach either with the PWD's carers or the PWD themselves (depending on dementia severity and/or living situation) to give them the Information Sheet and to discuss if they were willing to be approached by the researcher regarding this study. Participants who had no objection were contacted by the researcher by phone and were given more information about the study as required. In addition, details of people who had attended the centre (PWD's name, carer's name, address, phone, etc.) were provided and a letter was sent to the carer and/or patient including information sheets about the study. One week after, the researcher contacted them by phone, answered any questions they might have, explained the study and looked for their willingness to participate. If the potential participant agreed to be involved, either the researcher or an interviewer arranged a day to carry out the interview at their homes.

### Procedure

The study was carried out in accordance with the latest version of the Declaration of Helsinki. Ethical approval was granted by East London & the City HA Research Ethics Committee 3. All participants gave informed consent. Once the interviewer was at the PWD's or carer's home, they answered any further queries and sought informed consent as follows: written consent by signing a Consent Form was required from people with dementia and their caregivers. In order to achieve this, the interviewer approached potential subjects to explain the study and to inform them about their right to withdraw at anytime. Some PWD (11, 7.3%) were unable to provide written consent. When this occurred, the interviewer sought their assent (verbal consent). During this process, the interviewer made sure that he/she had taken as much time and care in explaining the information about this research as simply as possible. The interviewer avoided using long sentences and attempted to reduce any distractions. To find out if the participants have understood the information given, the interviewer observed their ability to ask any relevant questions and also requested the participant to repeat back the information and how it would relate to them. In addition, the interviewer clarified any doubts about the study and reiterated their right to withdraw at anytime.

Interviews were carried out by an experienced clinical psychologist and old age psychiatrists who were trained to undertake the interview. All the interviews with PWD were carried out at their homes. Interviews with carers were undertaken either at the PWD's home, their own home or at a health centre (e.g. memory clinic, day hospital). Whenever possible, the PWD and the carer were interviewed separately. However, some carers wanted to be present during the PWD's interview. In this situation, the interviewer emphasized the fact that he/she was interested in knowing both parties' views about the PWD's needs and quality of life; and, as they were unique human beings, both opinions do not necessarily have to be coincident and both were considered valid. Interviews were terminated immediately in presence of any sign of discomfort. In addition, if the conditions at home were not appropriate to undertake the interview with the carer, the interviewer arranged a next meeting in other location.

Using standardised instruments, PWD were interviewed about their needs, cognitive status and quality of life. This interview took an average of 30 minutes depending on dementia severity. Carers were interviewed about the PWD's sociodemographic details, needs, behavioural and psychological symptoms, functional status, quality of life, social networks, and services received. In addition, carers were assessed about their own sociodemographic details, depression, anxiety and burden. This interview took about two hours and a half.

### Measures

#### Instruments administered to the person with dementia

##### Camberwell Assessment of Need for the Elderly (CANE) [11,12]

The CANE is a comprehensive tool which offers a structured evaluation of needs in older people in 24 areas of social, psychological, physical, and environmental needs rated as no need, met need or unmet need. The CANE also asks about formal and informal support/services. The CANE has shown good levels of reliability (α = 0.99) and validity (correlated with the CAPE-BRS, r = 0.66; and the Barthel r = -0.53) [11]. It assesses the needs of older people from the perspective of the PWD, the carer, the staff and the researcher. Since ratings from the researcher were obtained for the complete sample (n = 152), in this article only researcher ratings were included. Researchers were trained by an expert (MO) to undertake the interviews using the Camberwell Assessment of Need for the Elderly (CANE) [12]. Pilot interviews were discussed and agreement in rating criteria was achieved. The CANE was selected because it has good psychometric properties and it has already been used to assess the needs of PWD [1,13,14].

##### Mini Mental State Examination (MMSE) [15]

This test assesses cognitive function including orientation, memory and attention. MMSE has been widely used in clinical and research practice [16]. The MMSE takes 5 to 10 minutes to administer. Its reliability (internal consistency) in community samples range from 0.54 to 0.77 and 0.96 in medical patients [17]. Regarding validity, MMSE has shown high correlations with several other test that measure different aspects of cognitive functioning ranging from 0.66 to 0.93 [17]. This brief screening tool was used to estimate the severity of PWD's cognitive impairment. This instrument was rated by the researcher in an interview with the person with dementia.

##### Quality of Life in Alzheimer's Disease (QoL-AD) [18]

The QOL-AD measures quality of life in PWD including areas such as physical health, energy, mood, living situation, memory, family, marriage, friends, self as a whole, ability to do chores around the house, ability to do things for fun, money and life as a whole. The scale allows both self-report ratings from the person with dementia and proxy-ratings from the caregiver. The QOL-AD has shown good levels of reliability and validity. In the original study, internal consistency was good (α = 0.88) [18]. Regarding convergent and divergent validity, QOL-AD showed a negative correlation with depression (-0.20) but no significant correlation was found with cognition (-0.09, p = 0.19) [19]. This measure was chosen because it is short and easy to administer, it assesses PWDs' and carers' perceptions about the person with dementia's quality of life, it can be used with people with MMSE scores as low as three [13] and it has been pointed out by the INTERDEM group as the instrument of choice to assess quality of life in PWD [20]. The QoL-AD PWD version was administered by interviewing the PWD and the carer version was self-administered by the carer.

#### Instruments administered to the carer to obtain information about the person with dementia

*Camberwell Assessment of Need for the Elderly (CANE) *[11,12]*(See above)*

*Quality of Life in Alzheimer's Disease (QoL-AD) *[18]*(See above)*

##### Neuropsychiatric Inventory (NPI) [21]

The NPI is a structured interview designed to assess a broad range of behavioural and psychological symptoms commonly encountered in PWD [21]. This tool has shown high internal consistency reliability for the frequency/severity product scores (α = 0.88) and for the specific severity (α = 0.87) and frequency (α = 0.88) ratings [22]. The NPI has been indicated by the INTERDEM group as the measure of choice for assessing behavioural and psychological symptoms in dementia because it assesses a wide range of behaviours, it has shown sensitivity to behavioural changes and its comprises the assessment of carer's stress generated by the symptoms [20]. The NPI was rated by the researcher in an interview with the carer.

##### Physical Self-maintenance Scale (PSMS) and Instrumental Activities of Daily Living Scale (IADL) [23]

The PSMS assesses functional status through a rating made by the person or an informant about the person's ability to perform basic activities of daily living independently. The IADL scale has been designed to evaluate more complex daily tasks which reflect environmental adaptation. The IADL scale has shown good validity and reliability (from α = 0.87 to α = 0.91) [23]. This tool has been extensively utilised by researchers and clinicians to assess ability for instrumental functions. The PSMS/IADL has been widely used, it is easy to complete and has been recommended by the INTERDEM group as a measure of choice in dementia care research [20]. The PSMS and IADL were rated by the researcher in an interview with the carer.

##### Practitioner Assessment of Network Typology (PANT) [4]

The PANT was developed to assess older people's support networks. The instrument comprises 8 items about three main features: availability of local close kin, level of involvement of family friends and neighbours, and the level of interaction with the community and voluntary groups. Thus, networks are characterised into five main types:

• Local family dependent support network. Includes close local family ties with a few peripheral friends and neighbours. It is a small network (1-4). Older people in this network generally live very near to or in co-residence with an adult child, they are less likely to be in good health, and their community involvement is low.

• Locally integrated support network. It is the most common and the most robust. This network is composed by local family, friends and neighbours; it is larger than average (+7) and implies high levels of community involvement. This network is related to the fewest risks.

• Local self-contained support network. Reliance mainly on neighbours and occasional contact with at least one relative more than five miles distant. This network is average size (5-6). Community involvement, if any, is low. Risks are associated with concern for privacy.

• Wider community focused support network. It is characterized by the absence of local kin. This network includes mainly friends living within 5 miles, some neighbours, and family living more than 50 miles away. The size of this network is larger than average (+8). Community involvement is usually high.

• Private restricted support network. It is associated with absence of local kin other than the spouse. This network is composed by relatives who live more than 50 miles away and is smaller than average. Usually comprises two subtypes: independent married couples and older people who have withdrawn or become isolated from local involvement. It includes minimal contact with neighbours and no community involvement. Members of this network are most at risk.

The PANT was rated by the researcher in an interview with the carer.

##### Client Service Receipt Inventory (CSRI) [24]

This instrument collects retrospective information about the patient and the carer such as accommodation, medication, income and expenditure, hospitalisation and services received by the patient during the last three months [24]. The CSRI has been widely used and has proved useful to assess care receipt service and the associated costs. The CSRI was rated by the researcher in an interview with the carer.

#### Instruments administered to the carer to obtain information about themselves

##### Hospital Anxiety and Depression Scale (HADS) [25]

This self-administered instrument is divided into two subscales: anxiety (HADS-A) and depression (HADS-D) each one including seven items [25]. Internal consistency reports vary for HADS-A from 0.68 to 0.93, and for HADS-D from 0.67 to 0.90. Sensitivity and specificity for both subscales is about 0.80. Concurrent validity has been reported between 0.60 to 0.80 [26]. This measure was chosen because it does not include somatic items (which are not recommended when assessing anxiety and depression in older people), it can be used with younger and older carers [27] and it has been used in previous studies of dementia carers [13,28]. The HADS was self-administered by the carer.

##### Zarit Burden Interview (ZBI) [29]

The Zarit Burden Interview is composed by 22 questions about the impact of the PWD's disabilities on the caregiver's life. For each item, caregivers are asked to indicate how often they have felt that way. Reliability has been estimated at 0.71 and 0.91, and validity has been estimated at 0.7 [30]. This measure was chosen because it has good psychometric properties, it is the most consistently used in research and it was developed specifically for carers of PWD [29]. The BI was self-administered by the carer.

### Data Analysis

Statistical analyses were undertaken using the SPSS 15.0 software package and AMOS 7.0 [31]. The significance level used was p < 0.05. Since needs (met and unmet) were not distributed normally, non-parametric tests were performed. For a better understanding of the results, when comparing groups, means instead of ranks are shown.

In order to identify predictors of unmet need, a stepwise multiple linear regression was performed. In addition, in order to test the theoretical model a path analysis was carried out using AMOS 7.0. For this analysis, only the main variables of this study were considered: living situation (alone vs. with others), behavioural and psychological symptoms (NPI score), services received, unmet needs, social networks, quality of life (rated by patients and carers) and carers' mental health (depression, anxiety and burden). Variables skewed ≥1 ('Unmet needs' and 'NPI Score') were transformed [32]. Maximum likelihood method was used to estimate the model. Chi-square statistic for the model was reported. A non significant χ^2 ^value indicates that the model does not occur by chance. The Normed Fit Index (NFI), and Comparative Fit Index (CFI) were reported. Values ≥ 0.90 indicate good fitting of the model [33]. Finally, the Root Mean Square Error of Approximation (RMSEA) which is sensitive to the number of estimated parameters in the model and penalizes the lack of parsimony was reported. A RMSEA value less than 0.05 with a narrow confidence interval (CI) denotes adequate parsimony [34].

## Results

### Participants

#### Demographics and Clinical Characteristics of People with Dementia

The demographics and clinical characteristics of PWD are shown on Table 1. The age of PWD ranged from 60 to 94 years (M = 79.2, s.d. 6.8). There were 74 (48.7%) males and 78 (51.3%) females. Women (M = 80.6, s.d. 6.1) were significantly older than men (M = 77.7, s.d. 6.1) (t (150) = -2.7, *p <*0.01). One-hundred and forty seven (98.7%) of the participants were white, only one was black (0.7%) or Asian (0.7%). Most were living in a house (119, 81.5%), and the rest were living either in a flat (16, 11%) or sheltered housing (which consists of self-contained unfurnished flats with the services of a manager or warden who lives on the premises or nearby and can be contacted through an alarm system if necessary) (11, 7.5%). Over a half (84, 55.3%) were married/living with a partner, 58 (38.2%) were widowed, and the remainder were either separated/divorced (7, 4.6%) or single (3, 2.0%). About one third of the sample was living alone (50, 32.9%) and the rest were living with others (102, 67.1%). One hundred and thirty seven (90%) PWD had a carer and 15 (9.9%) had no identifiable carer. Only one person with dementia was a carer himself (0.7%).

**Table 1:** Demographics and clinical characteristics of people with dementia

Characteristic		%/Mean(s.d.)	95% CI
Age (years)	60-64	2.6	
	65-79	42.8	
	80-94	54.6	
			
Gender	Male	48.7	
	Female	51.3	
			
Ethnicity	White	98.7	
	Black	0.7	
	Asian	0.7	
			
Marital Status	Single	2.0	
	Married/Living with a partner	55.3	
	Separated/Divorced	4.6	
	Widowed	38.2	
			
Living situation	Live Alone	32.9	
	Live with Others	67.1	
			
Cognitive Impairment	Severe (0-10)	14.7%	
	Moderate (11-20)	38.0%	
	Mild (> 21)	47.3%	
			
Functional Status		6.5 (3.8)	[5.97-8.03]
			
PWD's QoL rated by themselves		34.3 (7.0)	[33.05-36.65]
			
PWD's QoL rated by carers		28.6 (5.7)	[27.00-30.41]
			
BPSD		14.6 (14.7)	[10.57-18.43]

The total sample of PWD had a mean MMSE score of 19.13 (s.d. 7.2) indicating moderate cognitive impairment. Almost half of PWD (71, 47.3%) had a mild level of cognitive impairment (MMSE >21), 57 (38%) had moderate cognitive impairment (MMSE 11-20), and 22 (14.7%) had severe cognitive impairment (MMSE 0-10). Participants had a mild to moderate functional impairment as measured by ADL and IADL scales (M = 6.53, s.d. 3.8). The mean score on the NPI was 14.6 (s.d. 14.7). PWD had a mean of 34.3 (s.d. 7.0) for their quality of life and the mean was 28.6 (s.d. 5.7) for PWD's quality of life assessed by carers.

#### Demographics and Clinical Characteristics of Carers

Table 2 shows the demographics and clinical characteristics of carers. The age of the 128 carers ranged from 41 to 92 years, with a mean age of 65.9 (s.d. 13.1). Most of them were older people (67.5%), women (71.1%), and were married (89.8%). Eighty two were spouses (64.1%) and 39 (30.5%) were a son/daughter of the person with dementia. The majority (79, 66.9%) were caring for their relative 24 hours a day followed by 31 (26.2%) who spent from 4 to 20 hours a week looking after the person with dementia. Almost three quarters of the carers (94, 74%) were living with the care receiver.

**Table 2:** Demographics and clinical characteristics of carers

Characteristic		%/Mean(s.d.)	95% CI
Age (years)	40-64	46.3	
	65-89	52.8	
	90-100	0.8	
			
Gender	Male	28.9	
	Female	71.1	
			
Marital Status	Single	4.7	
	Married/Living with a partner	89.8	
	Separated/Divorced	3.9	
	Widowed	1.6	
			
Carer Relationship	Spouse	64.1	
	Children	30.5	
	Other relative	3.9	
	Friend	0.7	
			
Co-resident Carer	Yes	74.0	
	No	26.0	
			
Depression		6.1 (3.8)	[5.20-7.32]
			
Anxiety		7.8 (4.6)	[6.46-9.09]
			
Burden		33.2 (17.1)	[27.76-37.13]

The mean score for carer's depression, measured by the HADS-D, was 6.1 (s.d. 3.8, range 0-17) and 34 (35.4%) carers scored as depression cases (HADS-D > 7). For carer's anxiety, the mean score on the HADS-A was 7.8 (s.d. 4.6, range 0-20) and 48 (50.0%) carers were identified as anxiety cases (HADS-A > 7). Carers had an average score of 33.2 (s.d. 17.1, range 0-74) on the Zarit Burden Interview, indicating a high level of burden (> 24). Out of the 108 carers, 72 (66.7%) had high level of burden (ZBI score > 24).

### Needs

The mean of total number of needs was 10.0 (s.d 3.3, range 3-19), and of these 7.38 were met needs (s.d. 2.8, range 0-17) and 2.64 were unmet needs (s.d. 2.5, range 0-11). The frequency of CANE met and unmet needs by area are shown on Table 3. The most frequent met needs were memory (143, 94.1%), food (123, 80.9%), money (117, 77%), looking after home (115, 75.7%), drugs (97, 63.8%), physical health (96, 63.2%) and self-care (82, 53.9%). The most common unmet needs were daytime activities (77, 50.7%), company (60, 39.5%), psychological distress (47, 30.9%), eyesight/hearing (33, 22.0%), and accidental self-harm (which refers to inadvertent risk situations such as leaving the gas taps on, getting lost, etc) (23, 15.1%).

**Table 3:** Frequency (%) of CANE met, unmet and total needs

(n = 152)	Met Needs n (%)	Unmet Needs n (%)	Total Needs n (%)
Accommodation	9 (5.9)	12 (7.9)	21 (13.8)
Looking after home	115 (75.7)	13 (8.6)	128 (84.3)
Food	123 (80.9)	9 (5.9)	132 (86.8)
Self-Care	82 (53.9)	14 (9.2)	96 (63.1)
Caring for another	1 (0.7)	0 (0.0)	1 (0.7)
Daytime Activities	46 (30.3)	77 (50.7)	123 (81.0)
Memory	143 (94.1)	8 (5.3)	151 (99.4)
Eyesight/Hearing	39 (26.0)	33 (22.0)	72 (48.0)
Mobility	50 (32.9)	14 (9.2)	64 (42.1)
Continence	31 (20.4)	8 (5.3)	39 (25.7)
Physical Health	96 (63.2)	9 (5.9)	105 (69.1)
Drugs	97 (63.8)	11 (7.2)	108 (71.0)
Psychotic Symptoms	14 (9.2)	14 (9.2)	28 (18.4)
Psychological Distress	30 (19.7)	47 (30.9)	77 (50.6)
Information	34 (22.4)	12 (7.9)	46 (30.3)
Deliberate Self-Harm	3 (2.0)	8 (5.3)	11 (7.3)
Accidental Self-Harm	33 (21.7)	23 (15.1)	56 (36.8)
Abuse/Neglect	11 (7.3)	4 (2.6)	15 (9.9)
Behaviour	10 (6.6)	5 (3.3)	15 (9.9)
Alcohol	3 (2.4)	3 (2.4)	6 (4.8)
Company	17(11.2)	60 (39.5)	77 (50.7)
Intimate Relationships	4 (2.6)	12 (7.9)	16 (10.5)
Money	117 (77.0)	4 (2.6)	121 (79.6)
Benefits	13 (8.6)	1 (0.7)	14 (9.3)

Mean (SD)	7.4 (2.8)	2.6 (2.6)	10.0 (3.3)

### Factors associated with unmet needs

PWD who were not married (including those single, separated/divorced and widowed) had significantly more unmet needs (M = 3.6, s.d 3.0) than those who were (M = 1.9, s.d 1.8) (*U *= 1914, *p *< 0.01). Social network type was converted into two groups: low-community involvement (family dependent, local self-contained and private restricted network types) and high-community involvement (locally integrated and wider community focused network types). Thus, low-community involvement networks are small networks (1-6 members) with low levels of community involvement and composed of few close local family ties (generally only one) and a small number of friends and/or neighbours; whilst high-community involvement networks are larger networks (+7 members) characterised by the presence of friends, neighbours and, in some cases, local members of the family; and by the high levels of community involvement.PWD living in a low-community involvement network had significantly more unmet needs (M = 3.2, s.d 2.8) than those living in a high-community involvement network (M = 1.7, s.d 1.8) (*U *= 1281.5, *p *< 0.01). PWD who had higher scores on the NPI (r_s _= 0.53; p < 0.01), lower quality of life (rated by carers) (r_s_= -0.25; p < 0.01), and those who were cared for by a younger (r_s _= -0.22, *p *< 0.05) and anxious carer (r_s _= 0.22, p < 0.05) had significantly more unmet needs (See Table 4). Also those who were looked after by a son/daughter had significantly more unmet needs (M = 3.2, s.d 2.7) than those cared for by their spouses (M = 1.8, s.d 1.8) (*U *= 1131.5, *p *< 0.01). No association was found between the total number of services received by PWD and unmet needs (r_s _= 0.136; p = 0.31).

**Table 4:** People with dementia's and carer's clinical factors and association with unmet needs

People with dementia's factors	Statistic	p
Cognitive Status (MMSE)	r_s _= 0.10	0.22
Functional Status (ADL-IADL)	r_s _= 0.16	0.09
Behavioural and psychological symptoms (NPI)	r_s _= 0.53	<0.01**
QoL rated by themselves	r_s _= -0.15	0.09
QoL rated by carers	r_s _= -0.25	<0.01**

**Carer's factors**	**Statistic**	**p**

Depression (HADS-D)	r_s _= 0.15	0.14
Anxiety (HADS-A)	r_s _= 0.22	<0.05*
Burden Interview	r_s _= 0.09	0.33

**p < 0.01; * *p *< 0.05; r_s_= Spearman's Rho

### Predictors of unmet needs

In order to determine which variables were the best predictors of unmet needs, a stepwise multiple linear regression analysis using the sub-sample who completed all questionnaires included as predictors (n = 95) was carried out (See Table 5). Unmet needs was entered as the dependent variable and the variables that, in bivariate analyses, were significantly associated with unmet needs (PWD's marital status (married/other), PWD's living situation (alone/with others), social network group (high-community involvement/low-community involvement), behavioural and psychological symptoms (NPI score), carer's age, carer's type of relationship with the PWD (spouse/others), and carer anxiety (HADS-A score) were entered as independent variables. Multicollinearity was not present within the model. Higher number of unmet needs was predicted by: higher behavioural and psychological symptoms (NPI score) (p < 0.01); low-community involvement social network type (p < 0.01); and being looked after by, a younger (p < 0.01), and a more anxious carer (p < 0.05) (*F *= 15.2, p < 0.001; *R*^2 ^= 0.51)

**Table 5:** Predictors of unmet needs

Variables (n = 95)	Beta	p
Behavioural and psychological Symptoms (NPI)	0.41	< 0.001
Grouped Social Network Type (PANT) (low vs high community involvement)	-0.33	0.001
Carer Age	-0.28	0.005
Carer Anxiety	0.23	0.027

**Variance explained by model (R**^**2**^**) %**	51	
**Adjusted R**^**2**^**%**	47	
**F = **	15.2	
**p**	< 0.001	

**Variables Excluded from the Model**	Patient's marital status	
	Patient's living situation	
	Carer's type of relationship with the patient	

### Relationship between unmet needs, social networks and quality of life

In order to test the mediation effect of *unmet needs *between *social network *and *quality of life*, two mediation analyses [35] were performed: one considering *quality of life rated by carers *as dependent variable (DV), and the other using *self-rated quality of life *as DV (See Table 6).

**Table 6:** Mediation Analyses

Unmet Needs as Mediator between Social Network and Quality of Life rated by Carers					
		***Dependent variable: Quality of life rated by carers***			

*Regression 1*	*Independent Variable(s)*	*B*	*SE*	*t*	*p*

	Constant	27.065	1.240	21.828	0.000
	Social network	0.537	0.441	1.218	0.226

		***Dependent variable: Unmet needs***			

*Regression 2*	*Independent Variable(s)*	*B*	*SE*	*t*	*p*

	Constant	4.041	0.498	8.121	0.000
	Social network	-0.508	0.177	-2.874	0.005

		***Dependent variable: Quality of life rated by carers***			

*Regression 3*	*Independent Variable(s)*	*B*	*SE*	*t*	*p*

	Constant	29.671	1.453	20.424	0.000
	Social network	0.200	0.437	0.457	0.649
	Unmet needs	-0.736	0.235	-3.132	0.002

**Unmet Needs as Mediator between Social Network and self-rated quality of life**					

		***Dependent variable: Self-rated quality of life***			

*Regression 1*	*Independent Variable(s)*	*B*	*SE*	*t*	*p*

	Constant	31.810	1.529	20.807	0.000
	Social network	0.965	0.539	1.790	0.076

		***Dependent variable: Unmet needs***			

*Regression 2*	*Independent Variable(s)*	*B*	*SE*	*t*	*p*

	Constant	4.041	0.498	8.121	0.000
	Social network	-0.508	0.177	-2.874	0.005

		***Dependent variable: Self-rated quality of life***			

*Regression 3*	*Independent Variable(s)*	*B*	*SE*	*t*	*p*

	Constant	33.582	1.842	18.233	0.000
	Social network	0.767	0.547	1.403	0.163
	Unmet needs	-0.470	0.278	-1.694	0.093

In the first model, three regression analyses were performed; one in which *social network *was entered as the independent variable (IV) and *quality of life rated by carers *as the DV, another in which *social network *was treated as the IV and *unmet needs *(the presumed mediator) was treated as DV, and a last one in which *social network *and *unmet needs *were entered as IV with *quality of life rated by carers *as the DV. The amount of mediation (indirect effect) was calculated by subtracting the regression coefficient (*social network *to *quality of life rated by carers*) in the third regression (with *unmet needs *controlled) from the regression coefficient (*social network *to *quality of life rated by carers*, again) in the first regression (with *unmet needs *not controlled). The reduction in the regression coefficient from the first regression to the third regression when *unmet needs *was controlled was 0.537 - 0.200 = 0.337 which suggests a partial mediating effect. The Sobel test indicated that the direct effect of *social network *on *quality of life rated by carers *was significantly reduced when *unmet needs *was added to the equation Z = 2.11, p < 0.05. The same procedure was followed to determine if *unmet needs *acted as mediator between *social networks *and *self-rated quality of life*. The reduction in the regression coefficient from the first regression to the third regression when *unmet needs *was controlled was 0.965 - 0.767 = 0.198 which suggests a partial mediating effect. The Sobel test indicated that *unmet needs *does not significantly mediate the relationship between *social network *and *self-rated quality of life *(Z = 1.45, p = 0.14).

### Testing the theoretical model using path analysis

**Figure 1:**
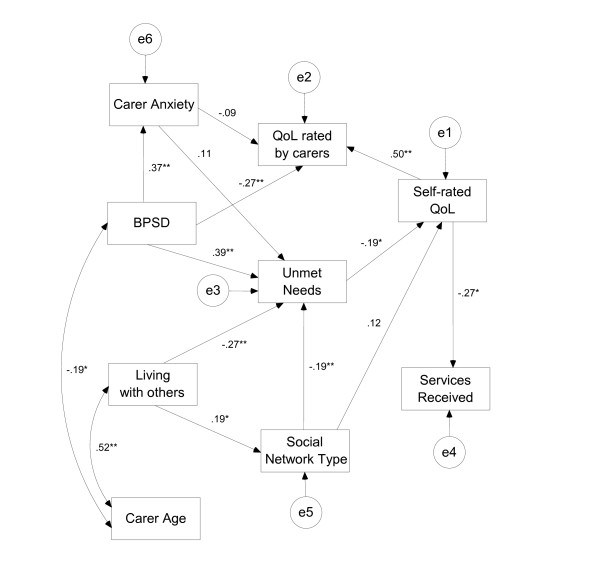
**Final Model**. Numbers indicate standardised regression coefficients (*p < 0.05; **p < 0.01); e = latent error.

The overall goodness of fit, for the hypothesized model, given by chi-square was χ^2 ^= 43.1, *df *= 17, *p *< 0.001. NFI was 0.67 and CFI was 0.73 indicating that the hypothesized model did not fit the data. After this result, an exploratory approach was adopted in order to construct a model that better reflects the data. The original model was modified following this process: Step 1: All the paths that had significant correlations were added to the hypothesized model. Regression paths were added from *behavioural and psychological symptoms *to *unmet needs*, from *behavioural and psychological symptoms *to *carer anxiety *(which was the only carer mental health factor significantly associated with unmet needs), from *carer anxiety *to *unmet needs*, from *carer anxiety *to *quality of life rated by carers*, from *carer age *(which in bivariate analyses was found associated with unmet needs) to *unmet needs*, from *self-rated quality of life *to *services received*, and the last one from *living with others *to *social network*. Two covariance paths (drawn from bivariate analyses) were also added, one between *carer age *and *living with others *and the second one between *carer age *and *behavioural and psychological symptoms*. The fit of the model after Step 1 was χ^2 ^= 9.28, *df *= 17, *p *= 0.66; NFI = 0.86; CFI = 1.0; RMSEA = 0.0, indicating that this model still did not fit the data. In order to obtain a model which fits the data, the next steps (2 and 3) consisted of removing non significant paths one by one, starting with the least significant. Step 2: The path from *carer age *to *unmet needs *was removed (χ^2 ^= 10.24, *df *= 18, *p *= 0.74; NFI = 0.90; CFI = 1.0; RMSEA = 0.0). Step 3: The path from *unmet needs *to *quality of life rated by carers *was removed. After removing this last path, the rise in χ^2 ^statistic for the model was more than the critical ratio (showing that the resulting model is significantly less good at fitting the data). The fit of the new model improved considerably (χ^2 ^= 11.79, *df *= 19, *p *= 0.89; NFI = 0.92; CFI = 1.0; RMSEA = 0.0), however, in order to obtain a more parsimonious model, the accepted criterion of removing one by one the paths which were not significant --starting with the least significant-- until the point when the rise in χ^2 ^statistic for the model was more than the critical ratio (showing that the resulting model is significantly less good at fitting the data) was used. Figure 1 shows the model that best fit the data (n = 152). The general goodness of fit was χ^2 ^= 13.4, *df *= 22, *p *= 0.92 indicating a good fit. NFI was 0.92 and CFI was 1.0 showing that the model fit was very good. Finally, the RMSEA index was 0.00, 90% CI (0.00-0.02) indicating also a parsimonious model. Having more behavioural and psychological symptoms (as measured by NPI), living alone and having a low-community involvement network were significant predictors of unmet needs. Higher levels of anxiety in carers were predicted by having more behavioural and psychological symptoms in PWD. A higher carer-rated quality of life was predicted by a higher self-rated quality of life and fewer behavioural and psychological symptoms. In addition, a higher self-rated quality of life was predicted by fewer unmet needs; and a higher self-rated quality of life was associated with a low use of services. Finally, behavioural and psychological symptoms, living situation (alone vs. with others), and social networks, all had indirect effects on self-rated quality of life through unmet needs, and also on carer-rated quality of life through self-rated quality of life.

## Discussion

This was the first study which used a theoretical model to illustrate how unmet needs, social networks and quality of life of PWD living at home were interrelated with each other and with the PWD's and carer's characteristics.

### Factors associated with unmet needs

The mean number of unmet needs of PWD in this research was higher than those assessed in sheltered housing [8] but it was lower than the unmet needs found for PWD in residential care [1]. This is congruent with the fact that whereas PWD living in sheltered housing are less dependent and might have better access to support, PWD in residential care had a higher prevalence of depression, anxiety, physical dependence, and behavioural problems [1]. Despite this, the most common unmet needs were the same as in residential care suggesting that PWD may have similar unmet needs in the community or in institutional care. The finding that PWD who had a low-community involvement network type had higher unmet needs than those who had a high-community involvement suggests that it is important to maintain community involvement for PWD for as long as possible. In the UK, the resources available for PWD and their carers have increased over the last years, although this is not necessarily synonymous with better coordination and appropriateness in the delivery of care [36]. Furthermore, for PWD who do not like to attend any kind of group, there are befriending services and outreach workers which provide a good opportunity to socialise, however the current availability of these in the UK is limited. The contact of PWD with the world outside the home (even if the person is visited at home) could be beneficial in several ways: increasing their involvement with the community, diminishing their psychological and social unmet needs and ultimately improving their quality of life.

The total number of services used by PWD was not associated with unmet needs. However, PWD who had attended a day hospital or a day centre over the last three months had fewer unmet needs than those who had not. Perhaps these services are more suitable for the needs of people living at home because they can offer support for several areas such as daytime activities, memory, and company. However, these services were not within the most used by PWD. There could be several reasons for this, such as the lack of need, lack of availability, lack of knowledge about their existence, lack of suitability for the carer, etc. In addition, PWD who had been visited by a social worker and those who had received home delivered meals had higher number of unmet needs. However, after controlling for living situation, this later association was explained by the fact that people who used home delivered meals were living alone [37].

Higher behavioural and psychological symptoms (NPI score) were associated with higher unmet needs. In line with Hancock's care home study [1], people with depression and dementia had significantly more unmet needs. Neil & Bowie [38] found that depression, agitation and anxiety were reported by family carers as the most distressing symptoms of dementia. Since the family is the main provider of support for the needs of PWD living at home and behavioural and psychological symptoms are highly correlated with carer stress and burden [39] it is not surprising that people having more behavioural and psychological symptoms were found to have significantly more unmet needs.

Consistently with previous studies, carers' ratings of quality of life were lower than those of PWD [3,18]. In the present study PWD with higher self-rated quality of life had fewer unmet needs, but this association was not significant. In addition, higher carer defined quality of life among PWD was associated with fewer unmet needs. In residential care, Hoe et al. [3] found that higher self-rated quality of life correlated with fewer unmet needs rated by researchers. In residential care, researcher ratings of unmet needs may be more likely to mirror PWD's views about their own quality of life due to the homogeneous environment for participants. In contrast, PWD living at home will have a more varied physical and social environment (e.g, activities, relationships, support) and so their own views of quality of life may well differ from the researchers assessment of needs.

Having high behavioural and psychological problems, a low-community involvement network, being cared for by a younger carer, and being looked after by an anxious carer explained half of the variability in unmet needs. Besides carer's age, all of these factors are modifiable. Thus, changing these factors would provide a chance to diminish the number of unmet needs. Psychosocial interventions and/or pharmacological interventions could be useful in diminishing neuropsychiatric symptoms [40]. In addition, Cooper et al. [41] have pointed out that psychosocial interventions aiming to diminish dysfunctional and increase emotion-focused coping could reduce carer's anxiety. The not explained variance in unmet needs may be caused, among other factors, by individual differences. The CANE allows the care practitioner to identify these individual unmet needs making possible the referral of each client to suitable interventions.

The mediation analyses showed that unmet needs were partially mediating the relationship between social networks and quality of life (self-rated or rated by carers); however the mediation effect was significant only for carer-rated quality of life. The PANT measures social networks from a structural perspective but does not provide any information about perceived social support, this is, the level of satisfaction with the help provided by the members of their social network. Thus, it might be that when PWD assess their quality of life, their perception of the quality of the support received may be more important than the structural aspect of their social network which may in turn perhaps be considered more by the carers when assessing the person with dementia's quality of life.

### Path analysis

The hypothesised model did not fit the data but an alternative model generated using the path analysis technique showed that people who had more developed social networks had significantly fewer unmet needs and, at the same time, those with fewer unmet needs had significantly higher self-rated quality of life. Thus, when rating their quality of life, PWD give more importance to the number of areas in which they are not receiving appropriate support (unmet needs) rather than to the structural aspects of their social network, even though these two variables are significantly associated with each other. Unmet needs may be reflecting the quality of care received which may have an impact on how PWD rate their own quality of life.

Improving the interventions and quality of care for dementia are two of the main issues highlighted by the National Dementia Strategy in the UK [42]. Therefore, the findings of this study have important implications for service providers. Behavioural and psychological symptoms, living situation, and social networks were associated with unmet needs and these are potentially modifiable. There are interventions available to reduce behavioural and psychological symptoms and to increase PWD's community involvement. Since changing living situation is difficult (it is unlikely that people living alone will shift to live with others, and that may not be necessarily beneficial), care providers should ensure that PWD living alone have the opportunity to receive any services that allow them to be in contact with the community, either by attending a psychosocial intervention (e.g. cognitive stimulation or reminiscence therapy) or social group; or by receiving support from a befriending visitor or outreach worker. Given their relevance to practice, it may be surprising that formalised assessment of social networks has not become part of case management. This could reflect reluctance on the part of practitioners to introduce standardised measures perhaps because it may be seen as increasing their workload and/or of little relevance to their everyday work.

### Methodological Considerations

Since the severity of the cognitive impairment could make difficult to contact PWD directly, whenever possible, the carer was also approached (either by phone or letter). However, this strategy resulted into two problems (sources of recruitment bias). First, in some cases the carer refused to participate on behalf of the person with dementia and consequently the person with dementia did not have the opportunity to be seen and asked by the researcher about their willingness to take part in the study. Second (either in absence of a carer or when the carer could not be reached) some PWD living alone who were not able to follow the conversation by phone and consequently could not agree to be interviewed, had to be excluded.

Even though carers were present in less than 10% of the interviews, PWD in this situation may have felt that they could not express their views freely, and consequently their answers may have been affected by the presence of the carer.

Since the interviews were long, the carers sometimes got tired and were unable to continue answering questions. Consequently, the last instrument (NPI) was sometimes not administered in order to prevent fatigue. The average interview took between two and a half to three hours. Interviews were carried out at the participant's own home (not in a clinical setting) which probably was more comfortable for the participants. If researchers had used the time only to get specific and brief answers to the questions, the interview would probably have been shorter. However, doing this would have not contributed to establish a good rapport with the person with dementia and carers. When carers got tired, it was useful to have a break and continue the interview after that. However, because of limited financial resources, it was generally not possible to visit people twice. In future research the interviews could be carried out in two sessions. Regarding the instruments, some of them could be replaced or omitted, or short versions could be used. For example, since social networks and use of services was measured by the PANT and CSRI respectively, it would be feasible to use only the short version of the CANE. Also, the CSRI could be shortened including only those services that are commonly used by PWD living in the community; however this could lead to problems estimating the overall costs.

### Limitations

In the regression analysis performed to determine which variables were the best predictors of unmet needs, "living situation" did not result in a significant predictor whereas in the path analysis the same variable came up as a significant predictor of unmet needs. This discrepancy may have been the result of the different sample size used for each analysis. While the regression was performed using only those participants who had complete data for all the predictors included in the model (n = 95), the path analysis was performed using maximum likelihood method, which dealt with missing data generating a model including the 152 participants. In addition, since a broad definition of carer was used, distinctions between those without a carer and those living with alone with an available carer were limited. Also, the regression model may have particularly excluded some people living alone who had no carer available. Therefore, the model generated using the path analysis should be considered as a better reflection of the relationship between living situation and unmet needs, indicating that living situation acts as a significant predictor of unmet needs. Higher behavioural and psychological symptoms (NPI score) were associated with a higher unmet needs but some of these symptoms assessed by the NPI are also included in the CANE (e.g. behaviour, psychological distress) which could have confounded these associations. However, the most frequent unmet needs of PWD (except for psychological distress) were found in areas not considered in the NPI such as, daytime activities, company, eyesight/hearing and accidental self-harm. The fact that only two people from minority ethnic groups was recruited, may be reflecting the difficulties these groups have in getting information about service availability and in accessing services in the areas where the recruitment was undertaken. Since this was a cross-sectional study, causality cannot be established from the results. Also, the final model generated from the path analysis was produced using an exploratory approach, so it has to be considered as a hypothetical model which needs to be tested in future research.

## Conclusions

PWD living at home had most of their physical and environmental needs met, but many social (daytime activities and company) and psychological (psychological distress and deliberate self-harm) were not receiving appropriate support. This confirms what literature about care management in dementia has already pointed out: first, the importance of systematically assessing to identify areas that would otherwise remain unrecognised; and second, the importance of having a coordinated interdisciplinary care management in order to provide services which target not only physical and environmental areas but also psychological and social ones [43,44].

The more important factors associated with unmet needs of PWD were BPSD and social network. The fact that the former is a clinical factor and the latter is a social one highlights the importance of treating dementia from an interdisciplinary perspective. PWD with a low-community involvement network had significantly more unmet needs than those with a high community involvement network type. According to the mediation and path analyses, social networks and behavioural and psychological symptoms have an indirect effect on PWD's quality of life through unmet needs. It would be interesting to develop an intervention which comprised the coordinated work of health and social services along with the voluntary sector. Thus, the management of PWD living at home would consider interventions aiming to decrease unmet needs by reducing behavioural and psychological symptoms (through pharmacological and/or psychosocial interventions) and involving PWD in the community (through attending to psychosocial interventions and/or social groups). By doing this, PWD's quality of life will be potentially improved. Finally, studying the effectiveness of this type of intervention would also provide the opportunity to test the model generated in this study.

## Competing interests

The authors declare that they have no competing interests.

## Authors' contributions

CMC designed the study, collected data, carried out the statistical analyses, wrote the paper, reviewed the manuscript and approved the final version. BW contributed to the interpretation of data, reviewed the manuscript and approved the final version. KG collected data, reviewed the manuscript and approved the final version. SO collected data, reviewed the manuscript and approved the final version. CO collected data, reviewed the manuscript and approved the final version. MO designed the study, contributed to the interpretation of data, reviewed the manuscript and approved the final version.
